# Supplementary material for: Antiparasitic Activity of Tea Tree Oil (TTO) and Its Components against Medically Important Ectoparasites: A Systematic Review
Source: Pharmaceutics. 2022 Jul 29;14(8):1587. doi: 10.3390/pharmaceutics14081587 (PMC9416580; doi:10.3390/pharmaceutics14081587)
Supplement: Supplementary file 1 [file pharmaceutics-14-01587-s001.zip › pharmaceutics-1714409-supplementary.pdf]

Supplementary Materials

Supplementary S1: Databases and search strategies

Supplementary Table S1. Full search strategy for articles on TTO and its components activity against ectoparasites

Databases	Step	Query	Item found
**PubMed**	#1	"Tea Tree Oil"[Mesh]	433
	#2	"Melaleuca"[Mesh]	223
	#3	#1 OR #2	582
	#4	"Ectoparasitic Infestations"[Mesh]	20,767
	#5	"Parasites"[Mesh]	7,261
	#6	"Mites"[Mesh]	17,014
	#7	"Mite Infestations"[Mesh]	7,053
	#8	"Scabies"[Mesh]	3,458
	#9	"Blepharitis"[Mesh]	1,324
	#10	"Pyroglyphidae"[Mesh]	3,051
	#11	"Trombiculidae"[Mesh]	632
	#12	"Pediculus"[Mesh]	1,143
	#13	"Lice Infestations"[Mesh]	2,715
	#14	"Phthiraptera"[Mesh]	3,050
	#15	"Flea Infestations"[Mesh]	776
	#16	"Siphonaptera"[Mesh]	3,816
	#17	"Tunga"[Mesh]	79
	#18	"Tungiasis"[Mesh]	115
	#19	"Bedbugs"[Mesh]	743
	#20	#4 OR #5 OR #6 OR #7 OR #8 OR #9 OR #10 OR #11 OR #12 OR #13 OR #14 OR #15 OR #16 OR #17 OR #18 OR #19	46, 917
	#21	#3 AND #20	**45**
**Embase via Scopus**	#1	TITLE-ABS-KEY "tea tree oil"	1,614
	#2	TITLE-ABS-KEY (“melaleuca alternifolia oil”)	59
	#3	#1 OR #2	1,624
	#4	TITLE-ABS-KEY (ectoparasites)	9,377
	#5	TITLE-ABS-KEY (“ectoparasitic infestations”)	2,872
	#6	TITLE-ABS-KEY (parasites)	266,246
	#7	TITLE-ABS-KEY (mites)	47,700
	#8	TITLE-ABS-KEY (“mite Infestations”)	4,236
	#9	TITLE-ABS-KEY (scabies)	7,738
	#10	TITLE-ABS-KEY (blepharitis)	3,915
	#11	TITLE-ABS-KEY (pyroglyphidae)	2,709
	#12	TITLE-ABS-KEY (trombiculidae)	991
	#13	TITLE-ABS-KEY (pediculus)	2,344
	#14	TITLE-ABS-KEY (“lice Infestations”)	2995
	#15	TITLE-ABS-KEY phthiraptera)	3063
	#16	TITLE-ABS-KEY (lice)	11,322
	#17	TITLE-ABS-KEY ("flea Infestations")	805
	#18	TITLE-ABS-KEY (siphonaptera)	4,211
	#19	TITLE-ABS-KEY (tunga)	424
	#20	TITLE-ABS-KEY (tungiasis)	461
	#21	TITLE-ABS-KEY (fleas)	10,564
	#22	TITLE-ABS-KEY (bedbugs)	978
	#23	#4 OR #5 OR #6 OR #7 OR #8 OR #9 OR #10 OR #11 OR #12 OR #13 OR #14 OR #15 OR #16 OR #17 OR #18 OR #19	340,611
	#24	#3 AND #23	**151**
**MEDLINE(EBSCOhost)**	S1	"tea tree oil"	666
	S2	“ectoparasitic infestations”	3,230
	S3	parasites	163,268
	S4	mites	25,122
	S5	“mite infestations”	10,636
	S6	scabies	4,680
	S7	blepharitis	2,010
	S8	pediculus	1,359
	S9	“lice infestations”	2,756
	S10	siphonaptera	3,617

	S11	"flea infestations"	707
	S12	bedbugs	881
	S13	S2 OR S3 OR S4 OR S5 OR S6 S7 OR S8 OR S9 OR S10 OR S11 OR S12	199,327
	S14	S1 AND S13	**70**
**CINAHL**	S1	"tea tree oil"	268
	S2	"ectoparasitic infestations" OR parasites OR mites OR "mite infestations" OR scabies OR blepharitis OR pediculus OR "lice infestations" OR siphonaptera OR "flea infestations" OR bedbugs	9,346
	S3	"tea tree oil" AND "ectoparasitic infestations" OR parasites OR mites OR "mite infestations" OR scabies OR blepharitis OR pediculus OR "lice infestations" OR siphonaptera OR "flea infestations" OR bedbugs	**26**
**Cochrane library (CENTRAL)**	#1	("tea tree oil") AND ("ectoparasitic infestations" OR mites OR "mite infestations" OR lice OR "lice infestations" OR blepharitis OR fleas OR "flea infestations" OR bedbugs) in Title Abstract Keyword - (Word variations have been searched)	**19**
**Web of Science**	#1	TS= ("tea tree oil")	1460
	#2	TS= ("melaleuca alternifolia oil")	56
	#3	#2 OR #1	1470
	#4	TS= (ectoparasites)	7,373
	#5	TS= ("ectoparasitic infestations")	40
	#6	TS= (parasites)	166,840
	#7	TS= (mites)	42,370
	#8	TS= ("mite Infestations")	242
	#9	TS= (scabies)	3,360
	#10	TS= (blepharitis)	1,288
	#11	TS= (pyroglyphidae)	293
	#12	TS= (trombiculidae)	559
	#13	TS= (pediculus)	754
	#14	TS= ("lice Infestations")	212
	#15	TS= (phthiraptera)	962
	#16	TS= (lice)	6,685
	#17	TS= ("flea Infestations")	146
	#18	TS= (siphonaptera)	1,651
	#19	TS= (tunga)	286
	#20	TS= (tungiasis)	303
	#21	TS= (fleas)	7,582
	#22	TS= (bedbugs)	405
	#23	#22 OR #21 OR #20 OR #19 OR #18 OR #17 OR #16 OR #15 OR #14 OR #13 OR #12 OR #11 OR #10 OR #9 OR #8 OR #7 OR #6 OR #5 OR #4	225,122
	#24	#23 AND #3	108
	#25	#23 AND #3 Refined by: [excluding] DOCUMENT TYPES: (REVIEW) I used this option	**80**
**ScienceDirect**	#1	("tea tree oil") AND (ectoparasites OR mites OR lice OR fleas OR bedbugs) limited to Research articles and Short communications	**87**
**SciELO**		("tea tree oil") AND (ectoparasites OR mites OR lice OR fleas OR bedbugs)	**1**
**LILACS**		("tea tree oil") AND (ectoparasites OR mites OR lice OR fleas OR bedbugs)- 53 (52 of them from MEDLINE and 1 review) so no new article from this database	0
**ATTI**		Tea tree oil	9
**Google**		Insecticidal and acaricidal activities of tea tree oil	9
**Total**			**497**

*During a complementary search on 04/04/22 12 articles were identified and included in systematic review.

Supplementary S2: PRISMA (Preferred Reporting Items for Systematic review and Meta-Analysis)

Supplementary Table S2: PRISMA (Preferred Reporting Items for Systematic review and Meta-Analysis) 2009 checklist

Section/topic	#	Checklist item	Reported on page #
**TITLE**			
Title	1	Identify the report as **a systematic review**, meta-analysis, or both.	1
**ABSTRACT**			
Structured summary	2	Provide a structured summary including, as applicable: background; objectives; data sources; study eligibility criteria, participants, and interventions; study appraisal and synthesis methods; results; limitations; conclusions and implications of key findings; systematic review registration number.	2
**INTRODUCTION**			
Rationale	3	Describe the rationale for the review in the context of what is already known.	2–3
Objectives	4	Provide an explicit statement of questions being addressed with reference to participants, interventions, comparisons, outcomes, and study design (PICOS).	3
**METHODS**			
Protocol and registration	5	Indicate if a review protocol exists, if and where it can be accessed (e.g., Web address), and, if available, provide registration information including registration number.	3
Eligibility criteria	6	Specify study characteristics (e.g., PICOS, length of follow-up) and report characteristics (e.g., years considered, language, publication status) used as criteria for eligibility, giving rationale.	3–4
Information sources	7	Describe all information sources (e.g., databases with dates of coverage, contact with study authors to identify additional studies) in the search and date last searched.	3–4
Search	8	Present full electronic search strategy for at least one database, including any limits used, such that it could be repeated.	Supplementary p1–2
Study selection	9	State the process for selecting studies (i.e., screening, eligibility, included in systematic review, and, if applicable, included in the meta-analysis).	4
Data collection process	10	Describe method of data extraction from reports (e.g., piloted forms, independently, in duplicate) and any processes for obtaining and confirming data from investigators.	4
Data items	11	List and define all variables for which data were sought (e.g., PICOS, funding sources) and any assumptions and simplifications made.	4
Risk of bias in individual studies	12	Describe methods used for assessing risk of bias of individual studies (including specification of whether this was done at the study or outcome level), and how this information is to be used in any data synthesis.	4–5
Summary measures	13	State the principal summary measures (e.g., risk ratio, difference in means).	Not applicable

Synthesis of results	14	Describe the methods of handling data and combining results of studies, if done, including measures of consistency (e.g., I^2) for each meta-analysis.	Not applicable
Risk of bias across studies	15	Specify any assessment of risk of bias that may affect the cumulative evidence (e.g., publication bias, selective reporting within studies).	Not applicable
Additional analyses	16	Describe methods of additional analyses (e.g., sensitivity or subgroup analyses, meta-regression), if done, indicating which were pre-specified.	Not applicable
**RESULTS**			
Study selection	17	Give numbers of studies screened, assessed for eligibility, and included in the review, with reasons for exclusions at each stage, ideally with a flow diagram.	5
Study characteristics	18	For each study, present characteristics for which data were extracted (e.g., study size, PICOS, follow-up period) and provide the citations.	9–34
Risk of bias within studies	19	Present data on risk of bias of each study and, if available, any outcome level assessment (see item 12).	35 & Supplementary p7–16
Results of individual studies	20	For all outcomes considered (benefits or harms), present, for each study: (a) simple summary data for each intervention group (b) effect estimates and confidence intervals, ideally with a forest plot.	9–34
Synthesis of results	21	Present results of each meta-analysis done, including confidence intervals and measures of consistency.	Not applicable
Risk of bias across studies	22	Present results of any assessment of risk of bias across studies (see Item 15).	Not applicable
Additional analysis	23	Give results of additional analyses, if done (e.g., sensitivity or subgroup analyses, meta-regression [see Item 16]).	Not applicable
**DISCUSSION**			
Summary of evidence	24	Summarize the main findings including the strength of evidence for each main outcome; consider their relevance to key groups (e.g., healthcare providers, users, and policy makers).	35–39
Limitations	25	Discuss limitations at study and outcome level (e.g., risk of bias), and at review-level (e.g., incomplete retrieval of identified research, reporting bias).	39
Conclusions	26	Provide a general interpretation of the results in the context of other evidence, and implications for future research.	39–40
**FUNDING**			
Funding	27	Describe sources of funding for the systematic review and other support (e.g., supply of data); role of funders for the systematic review.	40

Supplementary S3: Studies assessing AEs

Supplementary Table S3: A summary of studies reporting no AEs (n=12 no AEs)

Study	Study design	Treatment	Treatment schedule	AEs	Quality
Alver *et al*., 2017,[1] Turkey	Cohort study (*Demodex*)	TTO (10%) eyelash shampoo and TTO (4%) eyelid gel (n=28)	BID (Twice daily) for 1 month	No AEs	5 (Medium)
Epstein *et al*., 2020,[2] USA	Randomized double-blind, placebo-controlled trial	Cliradex® eyelid scrubs (T4O, no concentration reported) (n=26)	BID for 1 month	No AEs (the treatment was well tolerated and burning, or irritation symptoms reported by few patients (no specific number reported) dissipating in minutes or less)	5 (High)
Ergun *et al*., 2020,[3] Turkey	Randomized double-blind, placebo-controlled trial (*Demodex*)	TTO (3% w/w) plus < 5% (w/w) calendula oil, borage oil, vitamin E, vitamin B5 (n=25); TTO (3% w/w) gel (n=24)	BID for 1 month	No AEs	4 (High)
Gunnarsdóttir *et al*., 2016,[4] Iceland	Case study (*Demodex*)	Tea Tree wet wipes (*TTO concentration not stated*) (n=2)	BID for 10 weeks	No AEs	8 (High)
Karakurt and Zeytun, 2018,[5] Turkey	Randomised single-blinded controlled trial (*Demodex*)	TTO (7.5%) eyelash shampoo (n=75)	BID for 1 month	No AEs	2 (Low)
Koo *et al*., 2012,[6] South Korea	Randomized controlled trial (*Demodex*)	TTO (50%) lid scrub and TTO (10%) lid scrub (n=141)	TTO (50%) weekly and TTO (10%) daily for 1 month	4.7% (5/106) reported ocular irritation but disappeared following patient's education on the proper scrubbing method	2 (Low)
Mergen *et al*., 2021,[7] Turkey	Randomised double-blind, active comparator-controlled trial	TTO (7.5%) and chamomile oil (*no concentration reported*) swabs applied	BID for 2 months	No AEs	5 (High)
Tseng S. (NCT 01647217), 2017,[8] USA	Randomised controlled trial	T4O (Cliradex®) lid scrub (*no concentration reported*)	Once or BID for 1 month	No AEs	NA
Yam *et al*., 2014,[9] China	Case series (*Demodex*)	TTO (50%) lid scrub and tea tree shampoo (0.5 ml, TTO < 10 %) lid scrub (n=16)	TTO (50%) weekly and TTO shampoo BID for 3 weeks	No AEs	10 (High)
Wu *et al* 2019,[10] Chania	Quasi-experimental (*Demodex*)	TTO wipes (*concertation not reported*) and flurometholone (0.02%) eye drops (n=13); TTO wipes (*concertation not reported,* n=13)	BID for 1 month	No AEs	9 (High)
Whitledge 2002,[11] USA	Case study (Headlice)	TTO (9%) based shampoo (7% Anise oil and 4% lemon oil, 50% SD alcohol and 28% water & 2% fragrance)	One time application for 10–15 minutes	No AEs	7 (High)
Wong *et al*., 2019,[12] Australia	Randomised single blinded controlled pilot trial (*Demodex*)	Blephadex™ Eyelid Wipes (TTO and coconut oil, *concentration not reported*) (n=10)	Once daily for 1 month	No AEs	3 (High)

Supplementary Table S4: A summary of studies reporting no AEs (n=10)

Study	Study design	Treatment	Treatment schedule	AEs	Quality
Ebneyamin *et al*., 2019,[13] Iran	Randomized double-blind, placebo-controlled trial (demodex) (n=35 in both groups) (*Demodex*)	Permethrin (2.5%) with TTO (100%) gel (n=17)	BID for 3 months	No allergic reactions, and no major AEs observed but skin dryness (n=21, 60.0% moderate and 37.1% mild), burning and stinging (n=7, 20%), erosion (n=7, 20%) and erythema (n=3, 8.6%)	5 (High)
Liu and Gong, 2021,[14] China	Randomized controlled trial	TTO eye care patch (*no concertation reported*) (n=25)	Every night for 3 months	16% (4/25, slight to moderate irritation with conjunctival congestion	3 (High)
Maher 2018,[15] United Arab Emirates	Quasi-experimental (*Demodex*)	TTO (0.02%) eyelid (Naviblef™) scrub foam (n=20)	BID for 1 month	1 (contact dermatitis) in Test Vs 1 (eye irritation) in Control	9 (High)
Zulkarnain *et al*., 2019,[16] Indonesia	Randomized double blind controlled trial (Scabies)	TTO (5%) cream (n=24); TTO (5%) cream and permethrin (5%) cream (n=24); Permethrin (5%) cream	*No report on frequency of administration*	Minor irritation: *Week 1*: 0/24 in TTO group Vs 1/24 in Combination group Vs 1/24 in the permethrin group (*P=0.624*); *Week 2*: 6/24 in TTO group Vs 10/24 in Combination group Vs 2/24 in the permethrin group (*P=0.07*)	3 (High)
Gao *et al*., 2005,[17] USA	Cohort study (*Demodex*)	TTO (50%) lid scrub and tea tree shampoo (0.5 ml, TTO < 10 %) lid scrub (n=9)	TTO (50%) lid scrub weekly (three-time application) and tea tree (0.5 ml) shampoo daily (two time) for 1 month followed by once daily	TTO (50%) generated irritation in some patients (*no data is reported*)	9 (High)
Gao *et al*., 2007,[18] USA	Case series (*Demodex*)	TTO (50%) lid scrub and Tea Tree (0.5 ml, TTO < 10 %) shampoo lid scrub (n=11)	TTO (50%) lid scrub weekly (three-time application) and tea tree (0.5 ml) shampoo daily (two time) BID for 1 month	TTO (50%) office lid scrub caused mild irritation in 3 and moderate irritation in 6 participants	8 (High)
Gao *et al*., 2012,[19] USA	Cohort study (*Demodex*)	TTO (5%) ointment (n=24)	BID for 1 month	Mild ocular irritation in 2 participants	7 (High)
Barker and Altman, 2010,[20] Australia	Randomised assessor-blind controlled trial (Headlice)	TTO (10% w/v) / Lavender oil (LO, 1% w/v) lotion (n=43)	Three times on Days 1, 7 & 14	25 individuals with mild (n=22) and moderate (n=3) AEs (n=13 or 30.2% with stinging, n= 8 or 18.6% with flaky scalp/dry scalp and n=4 or 9.3% with erythema among these, n=3 moderate AEs (n=1, stinging of the eyes; n=1, stinging of the neck; and n=1, skin erythema) in TTO/LO group	5 (High)
Barker and Altman, 2011,[21] Australia	*Ex vivo* Randomised assessor-blind controlled trial (Headlice)	TTO (10% w/v) / LO (1% w/v) lotion (n=31)	Once on Days 1	4 (12.9%) individuals with mild AEs (n=3 stinging and n=1 redness) in TTO/LO group	5 (High)
Messaoud *et al*., 2019,[22] Tunisia	Randomized open level-controlled trial (*Demodex*)	Sterile wipe (T4O [2.5%] + hyaluronic acid [0.2%, moisturizing agent]) (n=24)	Once daily and BID for 29 days	1/24 (moderate burning sensation after application which resolved after 3s) in Test group I Vs 2/24 (visual acuity) in Test group II	2 (Low)

Supplementary S3: Quality assessments

Supplementary S3.1: Jadad Quality assessment for RCT

The methodological quality of included studies was assessed using the instrument developed by Jadad *et al.* (1996).[23] The scale awards one to five points to a RCT as presented in Supplementary table 4.

Supplementary Table S5: Criteria for quality assessment components of Jadad scale.

No	Items	Answers	Scoring
1.	Was the study described as randomized and method of randomization was sated?	Yes	+1
		No	0
2.	Was the method to generate the sequence of randomisation was described and it was appropriate?	Yes	+1
		Not described	0
		Inappropriate	-1
3.	Was the study described as double-blinding (participant and outcome assessor)?	Yes	+1
		No	0
4.	Was there adequate description of the method of masking (eg, identical placebo)?	Yes	+1
		No	0
		Inappropriate	-1
5.	Was there a description of withdrawals and dropouts for each group and If there were no withdrawals, is there a statement indicating no withdrawal?	Yes	+1
		No	0
**Total score**			5

Supplementary Table S6: Quality assessment results of the included studies using Jadad scale.

	**Author(s), Year**	**Randomisation 0–2 points**	**Blinding 0–2 points**	**Withdrawals 0–1 point**	**Total 0–5 points**
1.	Barker and Altman, 2010[20]	2	2	1	5
2.	Barker and Altman, 2011[21]	2	2	1	5
3.	Ebneyamin *et al*., 2019[13]	2	2	1	5
4.	Epstein *et al*., 2020[2]	2	2	1	5
5.	Ergun *et al*., 2020[3]	1	2	1	4
6.	Karakurt and Zeytun, 2018,[5]	1	0	1	2
7.	Koo *et al*., 2012[6]	1	0	1	2
8.	Liu and Gong, 2021[14]	2	0	1	3
9.	Mergen *et al*., 2021[7]	2	2	1	5
10.	Messaoud *et al*., 2019[22]	1	0	1	2
11.	Mohammadpour *et al.,* 2020[24] I	2	1	1	4
12.	Murphy *et al*., 2018[25]	1	0	1	2
13.	Tseng S. (NCT 01647217), 2017,[8] USA	-	-	-	NA
14.	Wang *et al*, 2020[26]	2	0	1	3
15.	Wong *et al*., 2019[12]	2	0	1	3
16.	Zhang *et al*., 2019[27]	1	0	1	2
17.	Zulkarnain *et al*., 2019[16]	1	1	1	3

NB: Tseng S. (NCT 01647217) is only available in registry record and could not evaluate the Jadad score.

Supplementary S3.2: JBI quality assessment for quasi-experimental studies

Supplementary Table S7: Criteria for JBI Quality assessment for quasi-experimental studies

No	Criteria
1.	Is it clear in the study what is the 'cause' and what is the 'effect' (i.e. there is no confusion about which variable comes first)?
2.	Were the participants included in any comparisons similar?
3.	Were the participants included in any comparisons receiving similar treatment/care, other than the exposure or intervention of interest?
4.	Was there a control group?
5.	Were there multiple measurements of the outcome both pre and post the intervention/exposure?
6.	Was follow up complete and if not, were differences between groups in terms of their follow up adequately described and analyzed?
7.	Were the outcomes of participants included in any comparisons measured in the same way?
8.	Were outcomes measured in a reliable way?
9.	Was appropriate statistical analysis used?

Supplementary Table S8: Quality assessment results of the quasi-experimental studies using JBI tool

	**Author(s), Year**	**1**	**2**	**3**	**4**	**5**	**6**	**7**	**8**	**9**	**Q**
1.	Maher 2018[15]	Y	Y	Y	Y	Y	Y	Y	N	Y	H
2.	Wu *et al* 2019[10]	Y	Y	Y	Y	Y	Y	Y	Y	Y	H
3.	Lu *et al*., 2021[28]	Y	Y	Y	Y	Y	Y	Y	Y	Y	H
4.	Zhong *et al*., 2021[29]	Y	Y	Y	Y	Y	Y	Y	Y	Y	H

Abbreviations: H: High; L: Low; M: Medium; Q: quality
Responses are reported as: Y: Yes; N: No; U: Unclear; NA: Not applicable

Supplementary S3.3: JBI Quality assessment for cohort studies

Supplementary Table S9: Criteria for JBI Quality assessment for cohort studies

No	Criteria
1.	Were the two groups similar and recruited from the same population?
2.	Were the exposures measured similarly to assign people to both exposed and unexposed groups?
3.	Was the exposure measured in a valid and reliable way?
4.	Were confounding factors identified?
5.	Were strategies to deal with confounding factors stated?
6.	Were the groups/participants free of the outcome at the start of the study (or at the moment of exposure)?
7.	Were the outcomes measured in a valid and reliable way?
8.	Was the follow up time reported and sufficient to be long enough for outcomes to occur?
9.	Was follow up complete, and if not, were the reasons to loss to follow up described and explored?
10.	Were strategies to address incomplete follow up utilized?
11.	Was appropriate statistical analysis used?

Supplementary Table S10: Quality assessment results of the cohort studies using JBI tool

	Author(s), Year	1	2	3	4	5	6	7	8	9	10	11	Q
1.	Alver *et al*., 2017[1]	NA	NA	Y	U	U	Y	Y	Y	N	N	Y	M
2.	Gao *et al*., 2005[17]	Y	Y	Y	U	U	Y	Y	Y	Y	Y	Y	H
3.	Gao *et al*., 2012[19]	NA	NA	Y	U	U	Y	Y	Y	Y	Y	Y	H
4.	Hirsch-Hoffmann *et al*., 2015[30]	NA	NA	Y	U	U	Y	N	Y	N	N	N	L
5.	Jacobi *et al*., 2021[31]	NA	NA	Y	Y	Y	Y	Y	Y	Y	Y	Y	H
6.	Kim *et al*., 2011[32]	NA	NA	Y	U	U	Y	Y	Y	Y	Y	Y	H

7.	Kojima *et al*., 2011[33]	NA	NA	Y	U	U	Y	Y	Y	Y	Y	Y	H
8.	Liang *et al*., 2018[34]	NA	NA	Y	Y	Y	Y	Y	Y	N	Y	Y	H
9.	McCage *et al*. 2002[35]	NA	NA	Y	U	U	Y	Y	Y	Y	Y	N	M
10.	Nicholls *et al*. 2016[36]	NA	NA	Y	Y	N	Y	N	Y	Y	N	N	M

Abbreviations: H: High; L: Low; M: Medium; Q: quality
Responses are reported as: Y: Yes; N: No; U: Unclear; NA: Not applicable

Supplementary S3.4: JBI Quality assessment for case series studies

Supplementary Table S11: Criteria for JBI Quality assessment for cohort studies

No	Criteria
1.	Were there clear criteria for inclusion in the case series?
2.	Was the condition measured in a standard, reliable way for all participants included in the case series?
3.	Were valid methods used for identification of the condition for all participants included in the case series?
4.	Did the case series have consecutive inclusion of participants?
5.	Did the case series have complete inclusion of participants?
6.	Was there clear reporting of the demographics of the participants in the study?
7.	Was there clear reporting of clinical information of the participants?
8.	Were the outcomes or follow up results of cases clearly reported?
9.	Was there clear reporting of the presenting site(s)/clinic(s) demographic information?
10.	Was statistical analysis appropriate?

Supplementary Table S12: Quality assessment results of the case series studies using JBI tool

	Author(s), Year	1	2	3	4	5	6	7	8	9	10	Q
1.	Evren Kemer *et al* 2020[37]	Y	Y	Y	N	N	Y	Y	Y	Y	Y	H
2.	Gao *et al*., 2007[18]	Y	Y	Y	U	U	Y	Y	Y	Y	Y	H
3.	Kheirkhah *et al*., 2007[38]	Y	Y	Y	U	U	Y	Y	Y	Y	U	H
4.	Liang *et al*., 2010[39]	Y	Y	Y	U	U	Y	Y	N	Y	U	M
5.	Patel *et al*. 2020[40]	Y	Y	Y	U	U	Y	Y	U	Y	U	M
6.	Yam *et al*., 2014[9]	Y	Y	Y	Y	Y	Y	Y	Y	Y	Y	H

Abbreviations: H: High; L: Low; M: Medium; Q: quality
Responses are reported as: Y: Yes; N: No; U: Unclear; NA: Not applicable

Supplementary S3.5: JBI Quality assessment for case studies

Supplementary Table S13: Criteria for JBI Quality assessment for cohort studies

No	Criteria
1.	Were patient's demographic characteristics clearly described?
2.	Was the patient's history clearly described and presented as a timeline?
3.	Was the current clinical condition of the patient on presentation clearly described?
4.	Were diagnostic tests or assessment methods and the results clearly described?
5.	Was the intervention(s) or treatment procedure(s) clearly described?
6.	Was the post-intervention clinical condition clearly described?
7.	Were adverse events (harms) or unanticipated events identified and described?
8.	Does the case report provide takeaway lessons?

Supplementary Table S14: Quality assessment results of the case studies using JBI tool

	Author(s), Year	1	2	3	4	5	6	7	8	Q
1.	Currie *et al*., 2004[41]	Y	Y	Y	Y	Y	Y	Y	Y	H
2.	Galea *et al*., 2014[42]	Y	Y	Y	Y	Y	Y	N	Y	H
3.	Gunnarsdóttir *et al*., 2016[4]	Y	Y	Y	Y	Y	Y	Y	Y	H
4.	Huo *et al*., 2021[43]	Y	Y	Y	Y	Y	Y	Y	Y	H
5.	Novelo 2015[44]	N	N	Y	N	N	Y	N	Y	L
6.	Tighe *et al*., 2013[45]	Y	N	Y	Y	Y	Y	N	Y	M
7.	Walton *et al*., 2004[46]	Y	Y	N	Y	Y	Y	N	Y	M
8.	Whitledge 2002[11]	Y	Y	Y	N	Y	Y	Y	Y	H
9.	Yin *et al*., 2021[47]	Y	Y	Y	Y	N	Y	N	Y	M

Abbreviations: H: High; L: Low; M: Medium; Q: quality
Responses are reported as: Y: Yes; N: No; U: Unclear; NA: Not applicable

Supplementary S3.6: ToxRTool assessment for in vitro studies

Supplementary Table S15: Criteria considered for ToxRTool reliability assessment for in vitro studies

No	Criteria
1.	Was the test substance identified?
2.	Is the purity of the substance given?
3.	Is information on the source/origin of the substance given?
4.	Is all information on the nature and/or physico-chemical properties of the test item given, which you deem indispensable for judging the data?
5.	Is the test system described?
6.	Is information given on the source/origin of the test system?
7.	Is necessary information on test system properties, and on conditions of cultivation and maintenance given?

8.	Is the method of administration given?
9.	Are doses administered or concentrations in application media given?
10.	Are frequency and duration of exposure as well as time-points of observations explained?
11.	Were negative controls included (give also point, if not necessary)?
12.	Were positive controls included (give also point, if not necessary)?
13.	Is the number of replicates (or complete repetitions of experiment) given?
14.	Are the study endpoint(s) and their method(s) of determination clearly described?
15.	Is the description of the study results for all endpoints investigated
16.	Are the statistical methods for data analysis given and applied in a transparent manner?
17.	Is the study design chosen appropriate for obtaining the substance-specific data aimed at?
18.	Are the quantitative study results reliable?

Supplementary Table S16: Quality assessment results of the in vitro studies using ToxRTool

	**Author(s), Year**	**1**	**2**	**3**	**4**	**5**	**6**	**7**	**8**	**9**	**10**	**11**	**12**	**13**	**14**	**15**	**16**	**17**	**18**	**Q**
1.	Bulut and Tanriverdi, 2021[48]	Y	Y	Y	Y	Y	Y	Y	Y	Y	Y	Y	Y	N	Y	Y	Y	Y	Y	R
2.	Cheung *et al*., 2018[49]	Y	Y	Y	Y	Y	Y	N	Y	Y	Y	Y	Y	Y	Y	Y	Y	Y	Y	R
3.	Frame *et al*., 2018[50]	Y	Y	Y	Y	Y	Y	N	Y	Y	Y	Y	Y	Y	Y	Y	Y	Y	Y	R
4.	Gao *et al*., 2005[17]	Y	Y	Y	Y	Y	Y	Y	Y	Y	Y	Y	Y	Y	Y	N	N	Y	Y	R
5.	Kabat 2019[51]	Y	Y	Y	Y	Y	Y	Y	Y	Y	Y	Y	Y	Y	Y	Y	Y	Y	Y	R
6.	Oseka and Sedzikowska, 2014[52]	Y	N	N	N	Y	Y	Y	Y	Y	Y	Y	Y	N	Y	Y	N	N	N	NA
7.	Tighe *et al*., 2013[45]	Y	Y	Y	Y	Y	Y	Y	Y	Y	Y	Y	Y	Y	Y	Y	Y	N	Y	R
8.	Yurekli and Botsali, 2021[53]	Y	Y	Y	Y	Y	Y	Y	Y	Y	Y	Y	Y	Y	Y	Y	N	Y	Y	R
9.	Fang *et al* 2016[54]	Y	Y	N	Y	Y	Y	Y	Y	Y	Y	Y	Y	Y	Y	Y	Y	Y	Y	R

	Author(s), Year	1	2	3	4	5	6	7	8	9	10	11	12	13	14	15	16	17	18	Q
10.	Walton *et al.,* 2000[55]	Y	Y	Y	Y	Y	Y	Y	Y	Y	Y	Y	Y	Y	Y	Y	Y	Y	Y	R
11.	Walton *et al*., 2004[46]	Y	Y	Y	Y	Y	Y	Y	Y	Y	Y	Y	Y	Y	Y	Y	Y	Y	Y	R
12.	Hill *et al*., 2001[56]	Y	Y	N	Y	Y	N	N	Y	Y	Y	N	Y	N	N	Y	N	N	N	NA
13.	McDonald and Tovey, 1993[57]	Y	Y	Y	Y	Y	N	N	Y	Y	Y	Y	Y	Y	N	Y	Y	Y	Y	R
14.	Priestley *et al*., 1998[58]	Y	N	N	Y	Y	N	Y	Y	Y	Y	Y	Y	Y	Y	Y	N	Y	Y	R
15.	Rim and Jee, 2006[59]	Y	Y	Y	Y	Y	Y	Y	Y	Y	Y	Y	Y	Y	N	Y	N	N	N	NR
16.	Williamson *et al*., 2007[60]	Y	N	N	Y	Y	Y	Y	Y	Y	Y	Y	Y	Y	Y	Y	N	Y	Y	R
17.	Yang *et al*., 2013[61]	Y	Y	Y	Y	Y	Y	Y	Y	Y	Y	Y	Y	Y	Y	N	N	Y	Y	R
18.	Akkad *et al*., 2016[62]	Y	Y	Y	Y	Y	Y	Y	Y	Y	Y	Y	Y	Y	Y	Y	N	Y	Y	R
19.	Candy *et al*., 2018[63]	Y	Y	Y	Y	Y	Y	N	Y	Y	Y	Y	Y	Y	N	N	Y	Y	Y	R
20.	De Wolff, 2008,[64]	Y	N	N	Y	Y	Y	Y	Y	Y	Y	Y	Y	N	Y	Y	N	Y	Y	RwR
21.	Di Campli *et al*., 2012[65]	Y	Y	Y	Y	Y	Y	N	Y	Y	Y	Y	Y	Y	Y	Y	N	Y	Y	R
22.	Downs *et al*., 2000[66]	Y	Y	Y	Y	Y	Y	Y	Y	Y	Y	Y	Y	N	N	N	N	Y	Y	RwR
23.	Heukelbach *et al*., 2008[67]	Y	Y	Y	Y	Y	Y	Y	Y	Y	Y	Y	Y	Y	Y	Y	Y	Y	Y	R
24.	McCage *et al*. 2002[35]	Y	N	N	N	Y	Y	Y	Y	Y	Y	N	Y	N	Y	N	N	Y	N	NR
25.	Priestley *et al*., 2006[68]	Y	Y	Y	Y	Y	Y	N	Y	Y	Y	Y	Y	Y	N	N	N	Y	Y	RwR

	Author(s), Year	1	2	3	4	5	6	7	8	9	10	11	12	13	14	15	16	17	18	Q
26.	Veal 1996[69]	Y	Y	Y	Y	Y	N	Y	Y	Y	Y	Y	Y	Y	N	Y	N	Y	Y	R
27.	Williamson *et al.*, 2007[60]	Y	N	N	Y	Y	N	N	Y	Y	Y	Y	Y	Y	Y	Y	N	Y	Y	RwR
28.	Yang *et al.*, 2004[70]	Y	Y	Y	Y	Y	Y	Y	Y	Y	Y	Y	Y	Y	N	Y	N	Y	Y	R
29.	De Wolff, 2008[64]	Y	N	N	Y	Y	Y	Y	Y	Y	Y	Y	Y	Y	N	Y	N	Y	Y	RwR
30.	Nair and Sasi, 2017[71]	Y	N	N	Y	Y	N	N	Y	Y	Y	N	Y	Y	N	Y	N	Y	Y	NR

Abbreviations: R: Reliable without restrictions; RwR: Reliable with restrictions; NR: not reliable; NA: Not assignable
Responses are reported as: Y: Yes; N: No

Supplementary S3.7: ToxRTool assessment for in vivo studies

Supplementary Table S17: Criteria considered for ToxRTool reliability assessment for *in vivo* studies

No	Criteria
1.	Was the test substance identified?
2.	Is the purity of the substance given?
3.	Is information on the source/origin of the substance given?
4.	Is all information on the nature and/or physico-chemical properties of the test item given, which you deem indispensable for judging the data?
5.	Is the species given?
6.	Is the sex of the test organism given?
7.	Is information given on the strain of test animals plus, if considered necessary to judge the study, other specifications?
8.	Is age or body weight of the test organisms at the start of the study given?
9.	For repeated dose toxicity studies only (give point for other study types): Is information given on the housing or feeding conditions?
10.	Is the administration route given?
11.	Are doses administered or concentrations in application media given?
12.	Are frequency and duration of exposure as well as time-points of observations explained?
13.	Were negative and positive controls included (give point also, when absent but not required)?
14.	Is the number of animals per group given?
15.	Are sufficient details of the administration scheme given to judge the study?

16.	For inhalation studies and repeated dose toxicity studies only (give point for other study types): Were achieved concentrations analytically verified or was stability of the test substance otherwise ensured or made plausible?
17.	Are the study endpoint(s) and their method(s) of determination clearly described?
18.	Is the description of the study results for all endpoints investigated transparent and complete?
19.	Are the statistical methods applied for data analysis given and applied in a transparent manner?
20.	Is the study design chosen appropriate for obtaining the substance-specific data aimed at?
21.	Are the quantitative study results reliable?

Supplementary Table S18: Quality assessment results of the in vivo studies using ToxRTool

	Author(s), Year	1	2	3	4	5	6	7	8	9	10	11	12	13	14	15	16	17	18	19	20	21	Q
1.	Fitzjarrell 1995[72]	Y	Y	N	Y	Y	Y	Y	N	Y	Y	Y	Y	Y	N	N	Y	N	N	N	Y	Y	NR
2.	Novelo, 2015[44]	Y	Y	N	Y	Y	N	Y	Y	Y	Y	Y	Y	Y	Y	Y	Y	Y	N	N	Y	Y	RwR

Abbreviations: R: Reliable without restrictions; RwR: Reliable with restrictions; NR: Not reliable
Responses are reported as: Y: Yes; N: No

Supplementary S4: Study characteristics of the excluded studies

Supplementary S4.1: Excluded studies

Supplementary Table S19: Studies excluded after full text review (n=21)

No	Author, year	Title	Reason for exclusion
**Excluded during the first search**			
1.	Chen *et al*., 2019[73]	Acaricidal activity of plant-derived essential oil components against Psoroptes ovis in vitro and in vivo	Veterinary ectoparasites
2.	Ebneyamin et al., 2019[74]	The efficacy and safety of permethrin 2.5% with tea tree oil gel on rosacea treatment: A double-blind, controlled clinical trial	Duplicate
3.	Ellse *et al*., 2013[75]	Control of the chewing louse Bovicola (Werneckiella) ocellatus in donkeys, using essential oils	Veterinary ectoparasites
4.	Ellse *et al*., 2016[76]	Essential oils in the management of the donkey louse, Bovicola ocellatus	Veterinary ectoparasites
5.	Ergun *et al*., 2019	Comparison of Efficacy and Safety of Two Tea Tree Oil-Based Formulations in Patients with Chronic Blepharitis: a Double-Blinded Randomized Clinical Trial	Duplicate
6.	James and Callander 2012a[77]	Dipping and jetting with tea tree (Melaleuca alternifolia) oil formulations control lice (Bovicola ovis) on sheep	Veterinary ectoparasites
7.	James and Callander 2012b[78]	Bioactivity of tea tree oil from Melaleuca alternifolia against sheep lice (Bovicola ovis Schrank) in vitro	Veterinary ectoparasites
8.	Lam *et al*., 2020[79]	Melaleuca alternifolia (tea tree) oil and its monoterpene constituents in treating protozoan and helminthic infections	Wrong study design (Review)
9.	LopatinaIu and Ermina 2014[80]	[Peculicidal activity of plant essential oils and their based preparations]	No full text access
10.	Mills *et al*., 2004[81]	Inhibition of acetylcholinesterase by Tea Tree oil	Wrong outcome
11.	Murphy *et al*., 2020[82]	The effect of lid hygiene on the tear film and ocular surface, and the prevalence of Demodex blepharitis in university students	Wrong patient population
12.	Neves *et al*., 2020[83]	The sensitivity of Demodex canis (Acari: Demodicidae) to the essential oil of Melaleuca alternifolia - an in vitro study	Veterinary ectoparasites
13.	Patel and Raju, 2013[84]	Ocular demodicosis	Wrong study design (Review)
14.	Sands *et al*., 2016[85]	Residual and ovicidal efficacy of essential oil-based formulations in vitro against the donkey chewing louse Bovicola ocellatus	Veterinary ectoparasites
15.	Sugathan and Martin, 2010[86]	Galenicals in the treatment of crusted scabies	Wrong intervention
16.	Talbert and Wall, 2012[87]	Toxicity of essential and non-essential oils against the chewing louse, Bovicola (Werneckiella) ocellatus	Veterinary ectoparasites
17.	Yam *et al*., 2013[88]	Ocular demodicidosis as a risk factor of adult recurrent chalazion	Duplicate
**Excluded during the second search**			
18.	Chen *et al*., 2021[89]	Crotamiton-loaded tea tree oil containing phospholipid-based microemulsion hydrogel for scabies treatment: in vitro, in vivo evaluation, and dermatokinetic studies	Wrong outcome
19.	Ngo., et al., 2018[90]	Short-Term Comfort Responses Associated with the Use of Eyelid Cleansing Products to Manage Demodex folliculorum.	Wrong outcome: RCT study, did not study activity of products against *Demodex* mites rather on patient comfort for various treatments on a single day
20.	Tharmarajah. and Coroneo, 2021[91]	Corneal Effects of Tea Tree Oil.	Wrong outcome
21.	Qiu *et al*., 2018[92]	Satisfaction and convenience of using terpenoid-impregnated eyelid wipes and teaching method in people without blepharitis	Wrong outcome
22.	Zarei-Ghanavati *et al*., 2021[93]	Comparison of the Effect of Tea Tree Oil Shampoo With Regular Eyelid Shampoo in Meibomian Gland Dysfunction Treatment	Wrong outcome: RCT study, did not study activity of products against Demodex mites rather on patient comfort for various treatments on a single day

Supplementary S4.2: Summary of studies for veterinary ectoparasites

Supplementary Table S20: Summary of the key data on veterinary ectoparasites

Summary of descriptive characteristics of studies exploring TTO against veterinary important ectoparasites (mites and lice, n=9)

**Study setting**	**Study design**	**Method/ Assay**	**Intervention**	**Outcome measure**	**Treatment outcome**
Chen *et al.*, 2019,[73] Belgium	*In vitro* (n=660 cattle mites, *P. ovis*)	In vitro: acaricide contact assay (immersion test): immersing the mites in test solutions and stereo-microscopic examination of their immobility 24 h post-immersion<br>Fumigation assay: placing a drop of test solutions at the bottom of a Petri dishes, followed by placing mites on a filter paper at the centre of the lid covered by the filter paper then closing the Petri dishes with the lid. Stereomicroscopic examination of mites for 150 mins in contact assay & 60 mins in fumigation assay	**Contact assay**<br>**Test:** 5.0, 2.5, 1.25, 0.63, 0.32 and 0.16 % of geraniol, eugenol, carvacrol and 1,8-cineol<br>**Control:** Liquid paraffin oil or mineral oil<br>**Fumigation assay:**<br><br>**Test:** droplet (15 µL) of 100% geraniol, eugenol, carvacrol and 1,8-cineol<br>**Control:** droplet (15 µL) paraffin oil or mineral oil<br>**Residual assay:**<br>**Test:** LC50 and LC90 obtained from contact assay used<br>**Control:** paraffin oil or mineral oil (1.5ml)	**LC50 (%) at 24 hrs**<br>Immobility of adult mites and a lack of reactions or persistent immobility within 1 min following stimulation with a needle were considered indications of death<br>**LT50 (min):**<br>Immobility of adult mites and a lack of reactions or persistent immobility within 1 min following stimulation with a needle were considered indications of death	**LC50 (%):** 0.56% for geraniol Vs 0.38 for eugenol Vs 0.26% for carvacrol Vs no activity for 1,8-cineol Vs no activity for control<br><br>**LT50 (min):**40 min for geraniol Vs 67 for eugenol Vs 24 for carvacrol Vs 35 for 1,8-cineol Vs no report for control<br><br>**LT100:** 90 min for geraniol Vs 150 for eugenol Vs 50 for carvacrol Vs 90 for 1,8-cineol Vs all viable after 160 minutes<br>(*No p-value is reported*)
Ellse *et al.*, 2013,[75] UK	*In vitro* (n=360 donkey chewing louse, *Bovicola ocellatus*)<br><br>In vivo (n=30 donkeys with chewing lice)	In vitro: Filter paper contact or non-contact assays: exposing the lice with the essential oils (contact) or their vapour (non-contact) for up to 24hrs or 2hrs & examining their mortality<br>In vivo: donkeys with the lice were sprayed with TTO, Lavender or vehicle only and checked for lice 2 weeks after the applications	**In vitro**:<br>**Test**: TTO (5% and 10%), Lavender oil (5% and 10%)<br>**Control I**: Vehicle (water +0.2% (v/v) Tween 80)<br>**Control II**: Silicone oil (5% and 10%)<br>In vivo:<br>**Test I**: TTO (5%) suspension sprayed (2mL/kg) once every 2 weeks for 1 month (n=10)<br>**Test II**: Lavender oil (5%) suspension sprayed (2mL/kg) once every 2 weeks for 1 month (n=10)<br>**Control**: Vehicle solution (water+0.2% Tween 80) sprayed (2mL/kg) once every 2 weeks for 1 month (n=10)	Louse mortality rate: from treatment to non-viability (immobility and absence of any movement with or without stimulation with pin)<br>Log reduction of louse number (in vivo)<br>AEs occurrence	**Mean Louse mortality rate (after 120 minutes, contact):** 90% for TTO Vs 90% for Lavender Vs 50% for Control I Vs 0% for Control II (P<0.05)<br>TTO (5%) Vs TTO (10%) and<br>Lavender (5%) Vs Lavender (10%) (P>0.05)<br>**Mean louse mortality rate (after 24hrs, contact):** 100% for TTO and Lavender groups<br>**Mean Louse mortality rate (after 2hrs for non-contact):** 80% for TTO Vs 80% for Lavender Vs 0% for Control I Vs 0% for Control II (P<0.05)<br>TTO (5%) Vs TTO (10%) and<br>Lavender (5%) Vs Lavender (10%) (P>0.05)<br>Reduction in louse number after 1 month: TTO Vs Lavender Vs Control (P>0.05)<br>**AEs**: No AES
Ellse *et al.*, 2016,[76] UK	*In vivo* (n=198 donkeys with chewing lice, *Bovicola ocellatus*)	Donkeys with the lice were sprayed with TTO, Lavender or vehicle only and checked for lice 2 weeks after the applications	In vivo:<br>**Test I**: TTO (5%) suspension sprayed (2mL/kg) once every 2 weeks for 1 month with every<br>**Test II**: Lavender oil (5%) suspension sprayed (2mL/kg) once every 2 weeks for 1 month<br>**Control**: Vehicle solution (water + 1% polyvinylpyrrolidone) sprayed (2mL/kg) once every 2 weeks for 1 month	Reduction in total louse counts after 1 month<br>AEs occurrence	**Reduction in total louse counts after 1 month (95%CI):** 78% (67.3–89.1%) for TTO Vs 78% (67.2–89.2%) for Lavender oil Vs 0% for Control (P<0.001)<br>78% (67.3–89.1%) for TTO Vs 78% (67.2–89.2%) for Lavender oil (P = 0.8)<br>**AEs:** No AEs
James and Callander 2012b,[78] Australia	*In vitro* (n= 420 sheep lice, *Bovicola ovis Schrank and n= blowfly Lucilia cuprina*)	Both Treated surface and wool assays<br>Fumigant assay	Treated surface assays<br>**Test**: TTO (1%, 5%, 10% and 20%)<br>**Control I**: Acetone<br>**Control II**: grapeseed oil<br>Wool assays<br>**Test**: TTO (0.5, 0.75 and 1%)	Louse mortality rate: from treatment to non-viability (immobility and absence of any movement with or	**Treated surface assays**<br>10.0 (0.0) for 1% Vs 10.0 (±5.8) for 5% Vs 13.3 (±6.7) for 10% and 20% for 10.0 (±5.8) Vs 6.7 (±3.3) Control I (P > 0.05)<br>96.7% (±3.3) for 10% Vs 100% for 20% TTO Vs 0% for Control II (P < 0.05)

			Fumigant assay<br>**Test**: TTO (0.5%, 1% and 2%)	without stimulation with pin)	3.3 (±3.3) for 0.1% Vs 3.3 (±3.3) for 0.5% Vs 6.7 (±3.3) for 1% Vs 0 for 2.5% Vs 0% Control II (P > 0.05)<br>**Wool assays**<br>100% for 1% TTO<br>**Fumigant assay**<br>100% for 0.5%, 1% and 2% TTO
James and Callander 2012a,[77] Australia	*In vivo* (n= 18 sheep lice, *Bovicola ovis Schrank and n= blowfly Lucilia cuprina*)	**Immersion dipping**<br>Sheep infested with lice were dipped into a test solution for 1 minutes and inspected for live lice for 20 weeks<br>**Jetting method**<br>4L test solutions were jetted over the sheep body and examined for live lice for 20 weeks	**Test I**: Treated with TTO (1%, n=6)<br>**Test II**: Treated with TTO (2%, n=6)<br>**Control**: No treatment (n=6)	**Immersion dipping**<br>Louse counts per 10 cm fleece parting Percent reduction<br>**Jetting**<br>Percent reduction	**Immersion dipping**<br>**Mean ±SD louse counts**<br>0 (100% reduction) (BL: 8.3 ±1.8)) for TTO (1%) Vs 0 (100% reduction) (BL: 8.4 ±1.8) for TTO (2%) Vs 8.2 ±3.0 (BL: 8.8±2.0) for Control<br>**Percent reduction**<br>100% for TTO (1%) Vs 100% for TTO (2%) Vs 6.8% for Control<br>**Percent reduction (Jetting)**<br>84.0% for TTO (1%) Vs 78.1% for TTO (2%) Vs 0% for Control (*no statistics reported*)
Magi *et al.*, 2006,[94] Estonia	*In vivo* (n=72 pigs with sarcoptic mange mites, *Sarcoptes scabiei var. suis*)	Essential oils were applied over the body infested with mites and examined for 4 weeks	**Test**: TTO, Garlic, Black pepper, Juniper, Citronella grass, Pennyroyal, Eucalyptus essential oils and Mugwort, Wormwood, Tansy and Hogweed plat extracts applied twice, with one-week interval<br>**Control**: no treatment	**Survival rate**	**Survival rate (%)**<br>1.45% for TTO Vs 10.65 for Garlic Vs 11.75 for Black pepper Vs 14.62 for Juniper Vs 4.82for Citronella Vs 6.75 for Pennyroyal Vs 10.69 for Eucalyptus Vs 18.22 for Mugwort Vs 10.98 for Wormwood Vs 8.82 for Tansy Vs 6.66 for Hogweed Vs 101.31 for Control (*no statistics reported*)
Neves *et al.*, 2020,[83] Brazil	*In vitro* (n=27 *Demodex canis* mites)	The products (200μL) applied on the mites placed on microscope slides and examined for viability using microscope for about 8hrs	**Test**: TTO (3.13%, 5.0% 6.25%, 12.5%, 25%, 50%, and 100%) (n=21)<br>**Control I:** Amitraz (ANZ, 12.5%, n=3)<br>Control II: Johnson's Baby Shampoo (JBS, n=3)	Mite survival time (MST): from treatment to non-viability (absence of chelicerae and tarsi movement)	**Mean ± SD MST in minuets**: 100.7±98.2 for TTO (3.3%) Vs 88.3±82.9 for TTO (5%) Vs 98.7±30.6 for TTO (6.25%) Vs 33.3±8.3 for TTO (12.5%) Vs 12.0±2.0) for TTO (25%) Vs 13.3±5.0 for TTO (50%) Vs 8.0±3.5 for TTO (100%) Vs 333.3±88.5 for ANZ Vs 470.7±60.0 for JBS (*no statistics reported*)
Sands *et al.*, 2016,[85] UK	*In vitro* (n=120, *Bovicola ocellatus* donkey chewing lice and n=120 eggs)	Filter paper contact bioassays: exposing the lice with the essential oils (800μL) & examining their mortality using dissecting microscope for 24hrs.<br><br>The ovicidal activities of the essential oils investigated following similar procedure over 12 days	Lice mortality test (24hrs)<br>**Test I**: TTO (5%)<br>**Test II**: Lavender oil (5%)<br>**Control (vehicle) I**: PVP (5% w/v)<br>**Control (vehicle) II**: SLS (5% w/v)<br><br>Ovicidal test (12 days)<br>**Test I:** TTO (5%)<br>**Test II**: Lavender oil (5%)<br>**Control (vehicle)**: coconut oil (5%)	Mortality rate: from treatment to non-viability (absence of movement of the legs, mouthparts, antennae or abdomen even when stroked with a dissecting needle)	**Mortality rate (Mean ± SD) of adult lice:**<br>100±0% for TTO Vs 100±0% for lavender Vs 50.0±18.0% for Control I Vs 30.0±11.0% for Control II (*statistics not reported*)<br>**Mortality rate (Mean ± SD) of nymphs:**<br>73.3±27.0% for TTO Vs 90.0±10.0% for lavender Vs 26.7±9.0% for Control I<br>96.7±3.0% for TTO Vs 100±0% for lavender Vs 20.0±0.0% for Control II (*statistics not reported*)<br>**Ovicidal test (hatchability)**<br>0% for TTO Vs 0% for lavender Vs 72±10% for Control (P<0.01)
Talbert and Wall, 2012,[87] UK	*In vitro* (n=150, *Bovicola ocellatus* donkey chewing lice)	Filter paper contact bioassays: exposing the lice with the essential oils (600μL) & examining their mortality using dissecting microscope for 5hrs.	**Test**; 0.5%, 1%, 3%, 5% and 10% (v/v) of TTO terpinen-4-ol (T4O, 3%), Camphor, Clove bud, Eucalyptus, Lavender, and Peppermint oils<br>**Control**: ethanol	LC50 %: from treatment to non-viability (absence of movement of limbs and gut and failure to respond when the abdomen was stroked with entomological pin or forceps) LT50 (min)	**LC50 % (mean ±95%CI)**<br>0.98% (0.6–1.5) for TTO Vs 8.6 (5.7–22.9) for Camphor Vs 1.23 (0.8–1.96) for Clove Vs 1.19 (0.6–1.96) for Eucalyptus Vs 0.76 (0.3–1.2) for Lavender Vs 1.24 (0.76–1.8) for Peppermint (P ≤ 0.05 for all except camphor)<br>**LT50** (min) **(mean ±95%CI)**<br>30.2 (6.2–56.3) minutes for TTO Vs >300 for Camphor Vs 60.8 (19.7–104.5) for Clove Vs 34.4 (0.8–76.5) for Eucalyptus Vs 39.3 (11.8–67.8) for Lavender Vs 68.7 (30.0–111.8) for Peppermint Vs 16.3 (8.5–24.3) for T4O (P ≤ 0.05 for all except camphor)

References

1. Alver O, Kivanc SA, Budak BA, Tuzemen NU, Ener B, Ozmen AT. A clinical scoring system for diagnosis of ocular demodicosis. *Med Sci Monit* 2017; **23**: 5862-9.
2. Epstein IJ, Rosenberg E, Stuber R, Choi MB, Donnenfeld ED, Perry HD. Double-masked and unmasked prospective study of terpinen-4-ol lid scrubs with microblepharoexfoliation for the treatment of *Demodex* blepharitis. *Cornea* 2020; **39**(4): 408-16.
3. Ergun SB, Saribas GS, Yarayici S, et al. Comparison of efficacy and safety of two tea tree oil-based formulations in patients with chronic blepharitis: A double-blinded randomized clinical trial. *Ocul Immunol Inflamm* 2020; **28**(6): 888-97.
4. Gunnarsdóttir S, Kristmundsson Á, Freeman MA, Björnsson ÓM, Zoëga GM. *Demodex folliculorum* a hidden cause of blepharitis. *Laeknabladid* 2016; **102**(5): 231-5.
5. Karakurt Y, Zeytun E. Evaluation of the efficacy of tea tree oil on the density of *Demodex* mites (Acari: Demodicidae) and ocular symptoms in patients with demodectic blepharitis. *J Parasitol* 2018; **104**(5): 473-8.
6. Koo H, Kim TH, Kim KW, Wee SW, Chun YS, Kim JC. Ocular surface discomfort and demodex: Effect of tea tree oil eyelid scrub in *Demodex* blepharitis. *J Korean Med Sci* 2012; **27**(12): 1574-9.
7. Mergen B, Arici C, Yildiz-Tas A, et al. Swabs containing tea tree oil and chamomile oil versus baby shampoo in patients with seborrheic blepharitis: A double-blind randomized clinical trial. *Eye Contact Lens* 2021; **47**(11): 604-10.
8. Tseng S. *Demodex* blepharitis treatment study (DBTS NCT01647217. 2017. https://clinicaltrials.gov/ct2/show/NCT01647217 (accessed December 16, 2019 2019).
9. Yam JCS, Tang BSF, Chan T-M, Cheng ACK. Ocular demodicidosis as a risk factor of adult recurrent chalazion. *Eur J Ophthalmol* 2014; **24**(2): 159-63.
10. Wu Y, Guo XH, Gui MF, et al. Infection of *Demodex folliculorum* and efficacy of tea tree essenial oil combined with fluorometholone for elderly patients with meibomian gland dysfunction. *Intern Eye Sci* 2019; **19**(9): 1566-9.
11. Whitledge KL. Use of essential oils to repel and treat head lice. Google Patents; 2002.
12. Wong K, Flanagan J, Jalbert I, Tan J. The effect of Blephadex™ Eyelid Wipes on *Demodex* mites, ocular microbiota, bacterial lipase and comfort: a pilot study. *Cont Lens Anterior Eye* 2019; **42**(6): 652-7.
13. Ebneyamin E, Mansouri P, Rajabi M, Qomi M, Asgharian R, Azizian Z. The efficacy and safety of permethrin 2.5% with tea tree oil gel on rosacea treatment: A double-blind, controlled clinical trial. *J Cosmet Dermatol* 2020; **19**(6): 1426-31.
14. Liu W, Gong L. Anti-demodectic effects of okra eyelid patch in *Demodex* blepharitis compared with tea tree oil. *Exp Ther Med* 2021; **21**(4): 338.
15. Maher TN. The use of tea tree oil in treating blepharitis and meibomian gland dysfunction. *Oman J Ophthalmol* 2018; **11**(1): 11-5.
16. Zulkarnain I, Agusni RI, Hidayati AN. Comparison of tea tree oil 5% cream, tea tree oil 5%+ permethrin 5% cream, and permethrine 5% cream in child scabies. *Int J Clin Exp Med Sci* 2019; **4**(6): 87-93.
17. Gao YY, Di Pascuale MA, Li W, et al. *In vitro* and *in vivo* killing of ocular *Demodex* by tea tree oil. *Br J Ophthalmol* 2005; **89**(11): 1468-73.
18. Gao YY, Di Pascuale MA, Elizondo A, Tseng SC. Clinical treatment of ocular demodecosis by lid scrub with tea tree oil. *Cornea* 2007; **26**(2): 136-43.
19. Gao YY, Xu DL, Huang l J, Wang R, Tseng SC. Treatment of ocular itching associated with ocular demodicosis by 5% tea tree oil ointment. *Cornea* 2012; **31**(1): 14-7.
20. Barker SC, Altman PM. A randomised, assessor blind, parallel group comparative efficacy trial of three products for the treatment of head lice in children--melaleuca oil and lavender oil, pyrethrins and piperonyl butoxide, and a "suffocation" product. *BMC Dermatol* 2010; **10**: 6.
21. Barker SC, Altman PM. An *ex vivo*, assessor blind, randomised, parallel group, comparative efficacy trial of the ovicidal activity of three pediculicides after a single application--melaleuca oil and

lavender oil, eucalyptus oil and lemon tea tree oil, and a "suffocation" pediculicide. *BMC Dermatol* 2011; **11**: 14.
22. Messaoud R, El Fekih L, Mahmoud A, et al. Improvement in ocular symptoms and signs in patients with *Demodex* anterior blepharitis using a novel terpinen-4-ol (2.5%) and hyaluronic acid (0.2%) cleansing wipe. *Clin Ophthalmol* 2019; **13**: 1043-54.
23. Jadad AR, Moore RA, Carroll D, et al. Assessing the quality of reports of randomized clinical trials: Is blinding necessary? *Control Clin Trials* 1996; **17**(1): 1-12.
24. Mohammadpour M, Maleki S, Khorrami-Nejad M. The effect of tea tree oil on dry eye treatment after phacoemulsification cataract surgery: A randomized clinical trial. *Eur J Ophthalmol* 2020; **30**(6): 1314-9.
25. Murphy O, O'Dwyer V, Lloyd-McKernan A. The efficacy of tea tree face wash, 1, 2-Octanediol and microblepharoexfoliation in treating *Demodex folliculorum* blepharitis. *Cont Lens Anterior Eye* 2018; **41**(1): 77-82.
26. Wang YQ, Li Y, Dai PF, Tian F, Wang J, Ni SS. Effect of tea tree oil eye patch combined with eyelid margin deep cleaning device in treating *Demodex* blepharitis. *Intern Eye Sci* 2020; **20**(10): 1791-6.
27. Zhang X, Song N, Gong L. Therapeutic effect of intense pulsed light on ocular demodicosis. *Curr Eye Res* 2019; **44**(3): 250-6.
28. Lyu Xue-Feng, Shen Jin-Xia, Chen Ya-Qiong. Optimized intense pulsed light combined with tea tree essential oil eye patch to treat *Demodex* blepharitis *Int J Ophthalmol* 2021; **21**(1): 178-81.
29. Zhong QL, Zhang KY, Chen YY, Wang SL, Chen SY. Treatment of mite associated ophthalmopathy in tropical areas. *China Tropical Medicine* 2021; **21**(9): 912-6.
30. Hirsch-Hoffmann S, Kaufmann C, Bänninger PB, Thiel MA. Treatment options for *Demodex* blepharitis: Patient choice and efficacy. *Klin Monatsbl Augenheilkd* 2015; **232**(4): 384-7.
31. Jacobi C, Doan S, Pavel V, Chiambaretta F, Kärcher T. Different approach to manage *Demodex* blepharitis - Initial and maintenance treatment. *Curr Eye Res* 2021: 1-9.
32. Kim JH, Chun YS, Kim JC. Clinical and immunological responses in ocular demodecosis. *J Korean Med Sci* 2011; **26**(9): 1231-7.
33. Kojima T, Ishida R, Sato EA, et al. *In vivo* evaluation of ocular demodicosis using laser scanning confocal microscopy. *Investig Ophthalmol Vis Sci* 2011; **52**(1): 565-9.
34. Liang L, Liu Y, Ding X, Ke H, Chen C, Tseng SCG. Significant correlation between meibomian gland dysfunction and keratitis in young patients with *Demodex brevis* infestation. *Br J Ophthalmol* 2018; **102**(8): 1098-102.
35. McCage CM, Ward SM, Paling CA, Fisher DA, Flynn PJ, McLaughlin JL. Development of a paw paw herbal shampoo for the removal of head lice. *Phytomedicine* 2002; **9**(8): 743-8.
36. Nicholls SG, Oakley CL, Tan A, Vote BJ. *Demodex* treatment in external ocular disease: the outcomes of a Tasmanian case series. *Int Ophthalmol* 2016; **36**(5): 691-6.
37. Evren Kemer Ö, Esra Karaca E, Özek D. Efficacy of cyclic therapy with terpinen-4-ol in *Demodex* blepharitis: Is treatment possible by considering *Demodex*'s life cycle? *Eur J Ophthalmol* 2020.
38. Kheirkhah A, Casas V, Li W, Raju VK, Tseng SCG. Corneal manifestations of ocular *Demodex* infestation. *Am J Ophthalmol* 2007; **143**(5): 743-9.e1.
39. Liang L, Safran S, Gao Y, Sheha H, Raju VK, Tseng SCG. Ocular demodicosis as a potential cause of pediatric blepharoconjunctivitis. *Cornea* 2010; **29**(12): 1386-91.
40. Patel NV, Mathur U, Gandhi A, Singh M. Demodex blepharokeratoconjunctivitis affecting young patients: A case series. *Indian J Ophthalmol* 2020; **68**(5): 745-9.
41. Currie BJ, Harumal P, McKinnon M, Walton SF. First documentation of *in vivo* and *in vitro* ivermectin resistance in *Sarcoptes scabiei*. *Clin Infect Dis* 2004; **39**(1): e8-12.
42. Galea M, Sharma R, Srinivasan S, Roberts F. *Demodex* blepharitis mimicking eyelid sebaceous gland carcinoma. *Clin Exp Ophthalmol* 2014; **42**(2): 208-10.
43. Huo Y, Mo Y, Jin X, Huang X, Chen W. First case of *Phthirus pubis* and *Demodex* co-infestation of the eyelids: A case report. *BMC Ophthalmol* 2021; **21**(1): 122.

44. Novelo C. Non-toxic ectoparasite treatment composition and method of use. Google Patents; 2015.
45. Tighe S, Gao YY, Tseng SCG. Terpinen-4-ol is the most active ingredient of tea tree oil to kill *Demodex* mites. *Transl Vis Sci Technol* 2013; **2**(7).
46. Walton SF, McKinnon M, Pizzutto S, Dougall A, Williams E, Currie BJ. Acaricidal activity of *Melaleuca alternifolia* (tea tree) oil: *In vitro* sensitivity of *Sarcoptes scabiei var hominis* to terpinen-4-ol. *Arch Dermatol* 2004; **140**(5): 563-6.
47. Yin HY, Tighe S, Tseng SC, Cheng AM. Successful management of chronic blepharo-rosacea associated *Demodex* by lid scrub with terpinen-4-ol. *Am J Ophthalmol Case Rep* 2021; **23**: 101171-.
48. Bulut A, Tanriverdi C. Anti-*Demodex* effect of commercial eyelid hygiene products. *Semin Ophthalmol* 2021: 1-4.
49. Cheung IMY, Xue AL, Kim A, Ammundsen K, Wang MTM, Craig JP. *In vitro* anti-demodectic effects and terpinen-4-ol content of commercial eyelid cleansers. *Cont Lens Anterior Eye* 2018; **41**(6): 513-7.
50. Frame K, Cheung IMY, Wang MTM, Turnbull PR, Waiters GA, Craig JP. Comparing the *in vitro* effects of MGO (TM) manuka honey and tea tree oil on ocular *Demodex* viability. *Cont Lens Anterior Eye* 2018; **41**(6): 527-30.
51. Kabat AG. *In vitro* demodicidal activity of commercial lid hygiene products. *Clin Ophthalmol* 2019; **13**: 1493-7.
52. Oseka MK, Sedzikowska A. Effect of essential oils on the survival rate of *Demodex* spp. *Investig Ophthalmol Vis Sci* 2014; **55**(13).
53. Yurekli A, Botsali A. The comparative *in vitro* killing activity of tea tree oil versus permethrin on *Demodex folliculorum* of rosacea patients. *J Cosmet Dermatol* 2021.
54. Fang F, Candy K, Melloul E, et al. *In vitro* activity of ten essential oils against *Sarcoptes scabiei*. *Parasites Vectors* 2016; **9**.
55. Walton SF, Myerscough MR, Currie BJ. Studies *in vitro* on the relative efficacy of current acaricides for *Sarcoptes scabiei var. hominis*. *Trans R Soc Trop Med Hyg* 2000; **94**(1): 92-6.
56. Hill N, Cameron M, Samah M. An evaluation of neem oil, tea tree oil and conventional insecticides to control house dust mites, the major indoor trigger of atopic allergies. Irvine: NeemCo; 2001. p. 47-9.
57. McDonald LG, Tovey E. The effectiveness of benzyl benzoate and some essential plant oils as laundry additives for killing house dust mites. *J Allergy Clin Immunol* 1993; **92**(5): 771-2.
58. Priestley CM, Burgess I, Williamson EM. Effects of essential oils on house dust mites. *J Pharm Pharmacol* 1998; **50**(S9): 193-.
59. Rim IS, Jee CH. Acaricidal effects of herb essential oils against *Dermatophagoides farinae* and *D. pteronyssinus* (Acari: Pyroglyphidae) and qualitative analysis of a herb *Mentha pulegium(pennyroyal)*. *Korean J Parasitol* 2006; **44**(2): 133-8.
60. Williamson EM, Priestley CM, Burgess IF. An investigation and comparison of the bioactivity of selected essential oils on human lice and house dust mites. *Fitoterapia* 2007; **78**(7-8): 521-5.
61. Yang JY, Cho KS, Chung NH, Kim CH, Suh JW, Lee HS. Constituents of volatile compounds derived from *Melaleuca alternifolia* leaf oil and acaricidal toxicities against house dust mites. *J Korean Soc Appl Biol Chem* 2013; **56**(1): 91-4.
62. Akkad DMHE, El-Gebaly NSM, Yousof H-ASA, Ismail MAM. Electron microscopic alterations in *Pediculus humanus capitis* exposed to some pediculicidal plant extracts. *Korean J Parasitol* 2016; **54**(4): 527-32.
63. Candy K, Nicolas P, Andriantsoanirina V, Izri A, Durand R. *In vitro* efficacy of five essential oils against *Pediculus humanus capitis*. *Parasitol Res* 2018; **117**(2): 603-9.
64. De Wolff R. Essential oil compositions for killing or repelling ectoparasites and pests and methods for use thereof. Google Patents; 2008.
65. Di Campli E, Di Bartolomeo S, Delli Pizzi P, et al. Activity of tea tree oil and nerolidol alone or in combination against *Pediculus capitis* (head lice) and its eggs. *Parasitol Res* 2012; **111**(5): 1985-92.

66. Downs AMR, Stafford KA, Coles GC. Monoterpenoids and tetralin as pediculocides [16]. *Acta Derm-Venereol* 2000; **80**(1): 69-70.
67. Heukelbach J, Canyon DV, Oliveira FA, Muller R, Speare R. *In vitro* efficacy of over-the-counter botanical pediculicides against the head louse *Pediculus humanus var capitis* based on a stringent standard for mortality assessment. *Med Vet Entomol* 2008; **22**(3): 264-72.
68. Priestley CM, Burgess IF, Williamson EM. Lethality of essential oil constituents towards the human louse, *Pediculus humanus*, and its eggs. *Fitoterapia* 2006; **77**(4): 303-9.
69. Veal L. The potential effectiveness of essential oils as a treatment for headlice, *Pediculus humanus capitis*. *Complement Ther Nurs Midwifery* 1996; **2**(4): 97-101.
70. Yang Y-C, Lee H-S, Clark JM, Ahn Y-J. Insecticidal activity of plant essential oils against *Pediculus humanus capitis* (Anoplura: Pediculidae). *J Med Entomol* 2004; **41**(4): 699-704.
71. Nair KPS, Sasi ARP. Tick and flea control compositions comprising plant essential oils and different forms for the ease of application. Google Patents; 2017.
72. Fitzjarrell EA. Method and formulation for eliminating fleas on animals. Google Patents; 1995.
73. Chen ZZ, van Mol W, Vanhecke M, Duchateau L, Claerebout E. Acaricidal activity of plant-derived essential oil components against Psoroptes ovis in vitro and in vivo. *Parasites Vectors* 2019; **12**(1).
74. Ebneyamin E, Mansouri P, Rajabi M, Qomi M, Asgharian R, Azizian Z. The efficacy and safety of permethrin 2.5% with tea tree oil gel on rosacea treatment: a double-blind, controlled clinical trial. *J Cosmet Dermatol* 2019.
75. Ellse L, Burden FA, Wall R. Control of the chewing louse *Bovicola (Werneckiella) ocellatus* in donkeys, using essential oils. *Med Vet Entomol* 2013; **27**(4): 408-13.
76. Ellse L, Sands B, Burden FA, Wall R. Essential oils in the management of the donkey louse, *Bovicola ocellatus*. *Equine Vet J* 2016; **48**(3): 285-9.
77. James PJ, Callander JT. Dipping and jetting with tea tree (*Melaleuca alternifolia*) oil formulations control lice (*Bovicola ovis*) on sheep. *Vet Parasitol* 2012; **189**(2-4): 338-43.
78. James PJ, Callander JT. Bioactivity of tea tree oil from Melaleuca alternifolia against sheep lice (Bovicola ovis Schrank) in vitro. *Vet Parasitol* 2012; **187**(3-4): 498-504.
79. Lam NS, Long X, Su XZ, Lu F. Melaleuca alternifolia (tea tree) oil and its monoterpene constituents in treating protozoan and helminthic infections. *Biomed Pharmacother* 2020; **130**.
80. Lopatina Iu V, Eremina O. [Peculicidal activity of plant essential oils and their based preparations]. *Med Parazitol (Mosk)* 2014; (2): 37-42.
81. Mills C, Cleary BJ, Gilmer JF, Walsh JJ. Inhibition of acetylcholinesterase by tea tree oil. *J Pharm Pharmacol* 2004; **56**(3): 375-9.
82. Murphy O, O' Dwyer V, Lloyd-McKernan A. The effect of lid hygiene on the tear film and ocular surface, and the prevalence of Demodex blepharitis in university students. *Cont Lens Anterior Eye* 2020; **43**(2): 159-68.
83. Neves R, Barros LA, Mendes SMC, et al. The sensitivity of *Demodex canis* (Acari: Demodicidae) to the essential oil of *Melaleuca alternifolia* - An *in vitro* study. *Rev Bras Parasitol Vet* 2020; **29**(3): e005220.
84. Patel KG, Raju VK. Ocular demodicosis. *W V Med J* 2013; **109**(3): 16-8.
85. Sands B, Ellse L, Wall R. Residual and ovicidal efficacy of essential oil-based formulations in vitro against the donkey chewing louse Bovicola ocellatus. *Med Vet Entomol* 2016; **30**(1): 78-84.
86. Sugathan P, Martin AM. Galenicals in the treatment of crusted scabies. *Indian J Dermatol* 2010; **55**(2): 195-7.
87. Talbert R, Wall R. Toxicity of essential and non-essential oils against the chewing louse, Bovicola (Werneckiella) ocellatus. *Res Vet Sci* 2012; **93**(2): 831-5.
88. Yam JCS, Tang BSF, Chan TM, Cheng ACK. Ocular demodicidosis as a risk factor of adult recurrent chalazion. *Eur J Ophthalmol* 2013; **24**(2): 159-63.

89. Chen L, Alrobaian M, Afzal O, et al. Crotamiton-loaded tea tree oil containing phospholipid-based microemulsion hydrogel for scabies treatment: in vitro, in vivo evaluation, and dermatokinetic studies. *Drug Deliv* 2021; **28**(1): 1972-81.
90. Ngo W, Jones L, Bitton E. Short-Term Comfort Responses Associated With the Use of Eyelid Cleansing Products to Manage Demodex folliculorum. *Eye & Contact Lens-Science and Clinical Practice* 2018; **44**: S87-S92.
91. Tharmarajah B, Coroneo MT. Corneal Effects of Tea Tree Oil. *Cornea* 2021; **40**(10): 1363-4.
92. Qiu TY, Yeo S, Tong L. Satisfaction and convenience of using terpenoid-impregnated eyelid wipes and teaching method in people without blepharitis. *Clin Ophthalmol* 2018; **12**: 91-8.
93. Zarei-Ghanavati S, Nooghabi MJ, Zamani G. Comparison of the Effect of Tea Tree Oil Shampoo With Regular Eyelid Shampoo in Meibomian Gland Dysfunction Treatment. *Am J Ophthalmol* 2021; **229**: 45-51.
94. Mägi E, Järvis T, Miller I. Effects of different plant products against pig mange mites. *J Acta Veterinaria Brno* 2006; **75**(2): 283-7.
